# Understanding the Sociocultural Challenges and Opportunities for Affordable Wearables to Support Poststroke Upper-Limb Rehabilitation: Qualitative Study

**DOI:** 10.2196/54699

**Published:** 2024-05-28

**Authors:** Rahat Jahangir Rony, Shajnush Amir, Nova Ahmed, Samuelson Atiba, Nervo Verdezoto, Valerie Sparkes, Katarzyna Stawarz

**Affiliations:** 1 School of Computer Science and Informatics, Cardiff University, Cardiff, United Kingdom; 2 Faculty of Electrical Engineering, Mathematics & Computer Science, University of Twente, Enschede, Netherlands; 3 Department of Electrical and Computer Engineering, North South University, Dhaka, Bangladesh; 4 The Future Laboratories, Ogbomoso, Nigeria; 5 School of Healthcare Sciences, Cardiff University, Cardiff, United Kingdom

**Keywords:** rehabilitation, wearables, upper-limb rehabilitation, user-centered design, qualitative, interviews, experiences, attitudes, perceptions, digital health, health technology, wearable, user centered design, design, home, stroke, recovery, affordable, low income, low resource, Bangladesh, physiotherapy

## Abstract

**Background:**

People who survive a stroke in many cases require upper-limb rehabilitation (ULR), which plays a vital role in stroke recovery practices. However, rehabilitation services in the Global South are often not affordable or easily accessible. For example, in Bangladesh, the access to and use of rehabilitation services is limited and influenced by cultural factors and patients’ everyday lives. In addition, while wearable devices have been used to enhance ULR exercises to support self-directed home-based rehabilitation, this has primarily been applied in developed regions and is not common in many Global South countries due to potential costs and limited access to technology.

**Objective:**

Our goal was to better understand physiotherapists’, patients’, and caregivers’ experiences of rehabilitation in Bangladesh, existing rehabilitation practices, and how they differ from the rehabilitation approach in the United Kingdom. Understanding these differences and experiences would help to identify opportunities and requirements for developing affordable wearable devices that could support ULR in home settings.

**Methods:**

We conducted an exploratory study with 14 participants representing key stakeholder groups. We interviewed physiotherapists and patients in Bangladesh to understand their approaches, rehabilitation experiences and challenges, and technology use in this context. We also interviewed UK physiotherapists to explore the similarities and differences between the 2 countries and identify specific contextual and design requirements for low-cost wearables for ULR. Overall, we remotely interviewed 8 physiotherapists (4 in the United Kingdom, 4 in Bangladesh), 3 ULR patients in Bangladesh, and 3 caregivers in Bangladesh. Participants were recruited through formal communications and personal contacts. Each interview was conducted via videoconference, except for 2 interviews, and audio was recorded with consent. A total of 10 hours of discussions were transcribed. The results were analyzed using thematic analysis.

**Results:**

We identified several sociocultural factors that affect ULR and should be taken into account when developing technologies for the home: the important role of family, who may influence the treatment based on social and cultural perceptions; the impact of gender norms and their influence on attitudes toward rehabilitation and physiotherapists; and differences in approach to rehabilitation between the United Kingdom and Bangladesh, with Bangladeshi physiotherapists focusing on individual movements that are necessary to build strength in the affected parts and their British counterparts favoring a more holistic approach. We propose practical considerations and design recommendations for developing ULR devices for low-resource settings.

**Conclusions:**

Our work shows that while it is possible to build a low-cost wearable device, the difficulty lies in addressing sociotechnical challenges. When developing new health technologies, it is imperative to not only understand how well they could fit into patients’, caregivers’, and physiotherapists’ everyday lives, but also how they may influence any potential tensions concerning culture, religion, and the characteristics of the local health care system.

## Introduction

### Background

Every year, more than 55 million people globally experience a stroke, which results in around 5 million deaths [1,2]. Those who survive the stroke may lose limb function in the upper limbs [3], which impacts motor control and can lead to long-term or permanent disability [2,4,5]. As a result, stroke patients cannot perform daily living activities such as eating, picking, and placing, and may become dependent on caregivers [4,6].

Stroke patients often undergo upper-limb rehabilitation (ULR) to improve their range of movements [7], which can help them lead independent lives and reduce reliance on caregivers. This rehabilitation is possible in hospital-based and home-based setups [8,9]. Traditional rehabilitation is conducted in a controlled environment in the hospital and includes action observation and mental imagery [8], task-specific training, and constraint-induced movement therapy with trained support personnel [9]. In contrast, home-based ULR focuses on everyday activities that reduce the requirement to visit hospitals [10]. However, access to rehabilitation can be an issue, especially in the Global South. For example, 97.25% of stroke patients in Bangladesh have limb weakness and require rehabilitation [3], and health inequalities mean that rehabilitation services are almost nonexistent [11,12]. Lack of rehabilitation or low engagement and compliance with it can lead to permanent disability, exacerbating poverty and inequality as people and their caregivers cannot work, creating a long-term dependency on caregivers [11]. Furthermore, patients often do not engage with home-based rehabilitation [13], may lose interest in repetitive exercises [14], or may incorrectly perform the exercises for fear of pain [7], negatively impacting the progress of their treatment. Factors such as low physical activity and self-efficacy, stress, lack of support, and adherence to physical treatment can further affect the treatment [7,15].

Novel technologies have been used to support rehabilitation in home-based settings, including virtual reality environments [16], wearable devices [17,18], or robotic devices for measuring upper-limb movements and improving the extension and flexion range of the arms [19-21]. Furthermore, electrical stimulation has been used to stimulate weak limbs [22]. However, these solutions are often complex, large, and expensive [23] and are difficult to integrate in everyday routine. As such, they are not appropriate for home use or low-income communities, especially in the Global South. Wearable technologies are a promising alternative, as they are small and can be worn at home. In recent years, several projects have explored the use of wearable devices to support rehabilitation [24-27] and patient monitoring [28], although their accuracy in identifying differences in upper-limb exercises is limited, and they have not been tested in the home environment. Therefore, there is a pressing need to develop affordable, low-cost ULR tools for stroke patients that support the integration of physiotherapy exercises within community health settings and at home to support recovery and increase the independence of stroke patients.

### Aims and Approach

This project aimed to gather contextual and design requirements for affordable, low-cost wearables to support poststroke ULR. In particular, we wanted to understand how ULR is perceived and practiced in Bangladesh and to compare the approach with the practice of physiotherapists from the United Kingdom. While these countries are economically different and are characterized by different cultures, understanding current rehabilitation practices in both settings and differences in approaches would highlight the unique needs of key stakeholders, including Bangladeshi physiotherapists and patients, and help to inform the design of low-cost ULR wearables.

As there is limited research on the user experience of rehabilitation devices in Global South settings (with most studies focused on the technical aspects, eg, Anowar et al [26]), we decided to follow the person-based approach [29] and prioritize understanding the needs of different stakeholder groups, as this is the first step in developing digital health interventions. By starting with qualitative research, we aimed to understand users’ experiences, their needs, and challenges they face when providing or receiving physiotherapy. This step is necessary when developing any new technologies or technology-based interventions as it allows researchers to identify a wide range of issues and discuss them in depth [29]. In our case, it would help to explore the challenges stroke patients face as a motivation to identify specific requirements for technology before spending time and resources on development [29]. Therefore, in this paper we report the results of interviews conducted with physiotherapists, caregivers, and patients.

## Methods

### Study Design

As this was the first step in the design process [29], the aim of the study was to understand the wider context within which users operate and to identify requirements for technology considering different stakeholders’ perspectives. Therefore, semistructured interviews were used as the main research method, as they help to understand a given topic in depth and allow researchers to ask follow-up questions while ensuring key topics are covered [30]. Furthermore, as they are a source of rich contextual data, fewer participants are required, especially when conducting an exploratory study with the aim to identify a broad range of related issues [30].

### Recruitment and Participants

We used a purposeful and targeted recruitment approach [31] to recruit representatives of all key stakeholder groups. We used our extended networks and local institutions to reach out to physiotherapists and recruited 4 Bangladeshi physiotherapists through medical colleges in Dhaka and 4 British physiotherapists through our contacts at the School of Healthcare Sciences at Cardiff University and the Stroke Association. Five of them were women, and 3 were men. They were aged between 35 and 50 years and had 8 to 14 years of experience working as physiotherapists; they all had experience with ULR. One British physiotherapist had an additional 14 years of experience as an academic.

Through Bangladeshi physiotherapists, we recruited 3 patients who underwent ULR in the past and 3 caregivers for people who had had a stroke. Patients were aged between 26 and 55 (SD 14.8) years; 2 were men. They underwent rehabilitation for stroke (male; 55 years), hand injury due to an accident (male; 35 years), and carpal tunnel syndrome (female; 26 years). We recruited 1 informal and 2 formal caregivers. The informal caregiver (a housewife) was recruited together with her husband (a patient). The 2 formal caregivers were recruited through formal phone calls to the Caregiver Institute in Bangladesh, where they both worked as caregiver trainers, while the informal caregivers received no such training. The caregivers were aged 40 to 55 years. Table 1 shows an overview of the participants.

**Table 1.** Overview of participants and types of sessions in which they participated (n=14).

Session type and participant ID		Participant type	Gender	Format	Country
**First round of individual interviews**					
	PT1	Physiotherapist	Female	Videoconference	United Kingdom
	PT2	Physiotherapist	Female	Videoconference	United Kingdom
	PT3	Physiotherapist	Female	Videoconference	United Kingdom
	PT4	Physiotherapist	Female	Videoconference	United Kingdom
**First group discussion**					
	PT5	Physiotherapist	Male	Videoconference	Bangladesh
	PT6	Physiotherapist	Female	Videoconference	Bangladesh
**Second group discussion**					
	PT7	Physiotherapist	Male	Videoconference	Bangladesh
	PT8	Physiotherapist	Male	Videoconference	Bangladesh
**Third group discussion**					
	P1	Patient	Male	Videoconference	Bangladesh
	C1	Caregiver	Female	Videoconference	Bangladesh
**Second round of individual interviews**					
	P2	Patient	Male	Videoconference	Bangladesh
	P3	Patient	Female	In person	Bangladesh
**Fourth group discussion**					
	C2	Caregiver	Male	In person	Bangladesh
	C3	Caregiver	Male	In person	Bangladesh

### Procedures

We conducted the interviews between March and October 2021. Given the physiotherapists’ busy schedules, they were given an option to attend individual or group sessions, depending on their preference and availability. Data were collected by 1 researcher in the United Kingdom and 3 researchers in Bangladesh. Semistructured interviews with physiotherapists were conducted remotely via Zoom (Zoom Video Communications, Inc) and lasted approximately 60 minutes. They were attended by 1 to 2 physiotherapists at the time; all British physiotherapists were interviewed individually, while 4 Bangladeshi physiotherapists joined in pairs. Regardless of the number of participants present, we followed the same protocol during both individual and group interviews.

After explaining the procedures and obtaining informed consent, the interviews started with questions about general experiences in delivering physiotherapy and the difficulties patients face. We then discussed standard practices in ULR following a stroke, focusing on exercises and movements that could be done at home and rehabilitation options available to patients after they leave the hospital. Finally, we talked about their current use of technology and the possibilities of developing a rehabilitation device, its features, and required factors for suitable home-based ULR.

Interviews with patients and caregivers were also semistructured and followed similar procedures; participants also had an option to attend an individual or a group interview and to decide whether they wanted to be interviewed in person or via videoconference. We interviewed 1 patient and their caregiver together via videoconference, 2 caregivers together in person, 2 patients individually via videoconference, and 2 others individually in person. When interviewing participants in person at their homes, we followed COVID-19 safety protocols, that is, we wore masks and maintained distance. Videoconference interviews were conducted through Zoom or Google Meet. The interviews covered similar topics to physiotherapist interviews: their experiences with rehabilitation, their preferences, and their use of technology in this context.

### Ethical Considerations

The research was approved by ethics committees at North South University and Cardiff University (COMSC/Ethics/2021/025). Consent forms for Bangladeshi participants were available in Bengali and English and for the British participants, only in English. British physiotherapists received £20 (US $15.08) shopping vouchers, while Bangladeshi participants received BDT 1000 (US $12) each for their participation; this discrepancy was dictated by the local rates and approved by the ethics committees.

### Analysis

The sessions with British physiotherapists were conducted in English and transcribed by a local transcription service. In contrast, Bangladeshi interviews were conducted in Bengali and then transcribed and translated by the researchers who collected the data. In total, we collected and transcribed about 10 hours of audio recordings, which resulted in a rich corpus of data comprising 87 pages (about 60,600 words). The analysis of both sets of interviews was conducted separately but followed the same procedures.

We used framework analysis [32] to analyze the data. The aims formed the basis of the framework used in the analysis of the physiotherapist interviews, and codes of interest included current approaches to physiotherapy, frequently used rehabilitation exercises, use of technology as part of the treatment, common barriers, and comments about a potential wearable system and its desired functionality. Then, based on reading the first few interview transcripts, the framework was updated and used to code the first 2 interviews from both the United Kingdom and Bangladesh. We used the web version of Atlas.ti (Atlas.ti Scientific Software Development GmbH) to code the transcripts, and coding was done by 1 member of the British research team and collaboratively by 3 members of the Bangladeshi research team. While coding the transcripts, we remained alert to potential insights and identified potential broader themes, which were then discussed by the research team during weekly meetings and incorporated into the final coding framework. Another member of the British research team then coded all British interviews, while the Bangladeshi team coded all of them; we then swapped and British team members reviewed the coded Bangladeshi transcripts and vice versa. After the coding was complete, we reviewed and summarized the content of each code and combined the ones with similar content. We then used the codes as columns in the framework table and the participants as rows, which enabled comparisons across the data and allowed us to identify themes.

We used a similar approach to analyzing patient and caregiver interviews, although in this case the coding guide for the physiotherapist interviews was used as a starting point and was adapted to accommodate codes unique to this participant group. All interviews were coded by the Bangladeshi team, who also summarized the framework table. The results were then discussed with the British team, and we identified the main themes together. Finally, we discussed all results to identify overarching themes, which we report in the next section.

## Results

### Overview

Our goal was to understand the rehabilitation practices and existing challenges of health professionals, patients, and caregivers. We also aimed to identify the contextual and design requirements for a low-cost wearable to support physiotherapy at home. We report 4 themes that have implications for remote therapy and developing rehabilitation devices for use at home.

### Theme 1: Sociocultural Factors Affecting Rehabilitation Practices

The interviews highlighted the impact of sociocultural practices on physiotherapy in Bangladesh. For example, access to therapy requires sensitive gendered consideration in Bangladesh, as varying genders of the physiotherapist and patient matter. As a result, families often discourage receiving support from a different gender, even if no other support is available:

*In Bangladesh, gender is another issue. Women do not take therapy from male therapists, and male patients do not take therapy from female therapists. Sometimes families discourage us from doing that. Older patients usually feel or consider the cultural barriers.*

[PT5, physiotherapist, man, Bangladesh]

Our results also showed that if physiotherapists and patients were of different genders, this could introduce additional unexpected barriers ranging from dismissal to potential harassment, which can negatively affect the treatment and discourage patients from engaging with rehabilitation or physiotherapists from attending certain patients. In addition, we noticed a widespread belief and clear expectations of what a physiotherapist should look like, with patients preferring physiotherapists of certain physical characteristics:

*Another perception in Bangladesh is physiotherapists should be healthy, tall, and stronger. So, I am small in size, which is why patients sometimes do not accept me. They openly express it, “How can you help with my movements?” And family members also tell us like, “Send someone healthy”.*

[PT6, physiotherapist, woman, Bangladesh]

Family support can also significantly impact the success of rehabilitation. For example, when family members help the patient too much with everyday activities, it can reduce their opportunities to engage in everyday actions that are beneficial to their overall rehabilitation and could discourage patients from engaging in formal exercises, hampering their independent movement in the long term. Both British and Bangladeshi participants mentioned this issue:

*I have worked with Indian communities around that area, and it was interesting that they did too much for their older people or people who were unwell. They do not let them do anything...their culture is to care for their elderly.*

[PT4, physiotherapist, woman, United Kingdom]

In addition, often the family’s religious beliefs have an impact on the rehabilitation process. For example, if the family strongly believes it is up to God whether someone will recover, they may discourage rehabilitation or not provide any support at home:

*Parents think if Allah wants, only then these kids can walk. They always ask us when their children can walk, but they don't cooperate. We always tell them that muscular dystrophy patients cannot walk, but they don't believe this. The mom of that family already works as a caregiver in a center, and should know this, but she never provides support to her baby.*

[PT6, physiotherapist, woman, Bangladesh]

However, despite potential barriers that family can introduce, it also plays an essential role. Participants from both countries reported that family members often helped with rehabilitation exercises or made sacrifices to enable the treatment. For example, one caregiver reported:

*At the beginning* [of the COVID-19 pandemic]*, his elder brother massaged him for around 2 hours daily.*

[C1, informal caregiver, woman, Bangladesh]

### Theme 2: Dimensions of Physiotherapy Practices in Rehabilitation

We also identified differences in the approach to therapy. The interviews with British physiotherapists revealed that they often took a holistic view of the treatment. They reported focusing not just on the immediate movements related to ULR but the broader context in which the patient operates, including functional movements (eg, completing everyday tasks such as getting dressed or eating), their mental health, and their general buy-in and understanding of the need for treatment.

*I think you would get disappointed if you were to aim at improving wrist flexion, for argument’s sake. When it’s the whole quality of life, you want to look at. So, it’s making it more holistic.*

[PT2, physiotherapist, woman, United Kingdom]

In contrast, Bangladeshi physiotherapists came across as more pragmatic by focusing on ensuring the patient had the building blocks needed for functional movements further down the line. For example, they emphasized focusing on a few significant movements, such as flexion, pronation, extension, and supination for the wrist, elbow, and shoulder. They also encouraged simple exercises like pinching to help activate muscles.

*We do an exercise such as grabbing a page sheet with two fingers together and pulling it. Stroke patients’ muscles don't have enough strength to do it. They are called intrinsic muscles; through this exercise, we activate them. If you can put the sensor in the fingertip, it is good.*

[PT5, physiotherapist, man, Bangladesh]

Therefore, Bangladeshi physiotherapists seemed less concerned by patients’ buy-in and expected them to practice the exercises, even if they involved repetitive movements. While they understood the benefits of holistic treatment, they preferred to focus on quick wins and targeted treatment to facilitate engagement. This was seen as more practical and helped regularly assess the progress of the patient, as it could be matched with their muscle power grades.

*In Stroke patients’ rehabilitation, the movements we are following depend on several stages with several movements. It depends on muscle power. When muscle power is 0, that means the patient is completely paralyzed. This time we do the movements for the paralyzed patient. We have a total of 6 grades: 0-5. In grade 1, the patient can move a bit. Grade 2 is similar but has better movement than grade 1. In grade 3, the patient can move hands against gravity a bit. In 4 and 5 grades, patients can move their hands far better. This time they do not require help.*

[PT7, physiotherapist, man, Bangladesh]

### Theme 3: Challenges of Home-Based Rehabilitation During and Beyond the Pandemic

While we were not explicitly interested in the impact of the COVID-19 pandemic on rehabilitation, it was impossible to ignore it, as it has exacerbated existing challenges to providing physiotherapy at patients’ homes and introduced new ones. Our participants highlighted issues related to movement accuracy, repetition, and COVID-19 contamination risks related to home-based support.

*During the lockdown, our centers were closed*.... *We are now trying to give home service so patients can at least continue the therapy at home. However, patients also do not allow physios to their homes due to COVID-19. Therefore, they can't take therapy and get negatively impacted.*

[PT6, physiotherapist, woman, Bangladesh]

Caregivers also reported that patients and people they looked after were hesitant to meet with physiotherapists due to COVID-19 concerns, both at the rehabilitation center and at home. For example, 1 informal caregiver shared her patients’ distrust and fear of catching the virus, which stopped them completely from engaging in physiotherapy:

*Physios move around. They will not treat only a single patient. That is why we feared COVID infection because my patient was vulnerable, and he still is. We tried to keep ourselves safe as much as possible. If COVID were not there, the treatment would go better.*

[C1, informal caregiver, woman, Bangladesh]

As the rehabilitation had to be delivered at home during the pandemic, it increased costs and further reduced the affordability in Bangladesh (“The cost was double or thrice for the home service.” [C1, informal caregiver, woman, Bangladesh]). As a result, our participants reported strategies that required balancing the affordability of the treatment with its effectiveness, such as bypassing physiotherapists and hiring nonprofessionals in their community to support physiotherapy at home:

*The same things happen in the house also. A maid does the movements they observe from therapists. So, the family discourages the therapists from coming home and paying a small amount to the maid* [nonprofessional] *to do the movements. This is bad for accuracy*.

[PT6, physiotherapist, woman, Bangladesh]

Apart from potential COVID-19 issues, unsupervised rehabilitation at home in general poses several risks. For example, our participants highlighted the risk of patients overdoing their exercises when practicing on their own. This may happen when they want to leave physiotherapy centers early and continue the exercises repeatedly without experts’ opinions. Furthermore, the physiotherapists explained that inaccurate movements, done without regular supervision, could hamper recovery or even lead to negative outcomes:

*When the patient can walk somehow at home, all are happy*...*this patient can completely get well if he is treated by an expert. That is why, the movement should be accurate, and otherwise the postures will be permanently changed for the patient.*

[PT6, physiotherapist, woman, Bangladesh]

In addition, home-based rehabilitation is often overseen by informal caregivers, usually family members. However, due to their lack of expertise, they may incorrectly support the movements, or patients may misunderstand what they are supposed to be doing if they rely on video prompts, which also can have negative long-term consequences.

### Theme 4: Attitudes Toward Rehabilitation Technologies

There was a clear difference in familiarity and exposure to rehabilitation technologies among the physiotherapists in the United Kingdom and Bangladesh. The British physiotherapists mentioned a wide range of rehabilitation devices they use at work, including rehabilitation gloves and functional electric stimulation. They also reported that, in general, patients liked using gadgets, which improved motivation and engagement:

*Saebo Glove helps to increase that movement and from a functional point of view, being able to use that glove around the house, it was a lot more helpful because you could use it in function with that little bit of extra help.*

[PT1, physiotherapist, woman, United Kingdom]

In contrast, Bangladeshi physiotherapists said they did not use or have wearable solutions, although they did use electrical rays and stimulators to stimulate muscles and nerves. At the same time, both caregivers and patients reported their interest in using wearables in rehabilitation. For example, C2, a professional caregiver trainer, explained that a wearable system with feedback would ease the activities of caregivers and therapists. Patients also shared the potential of using wearables that might detect wrong movements and provide feedback, which would improve movement accuracy. They also believed that it would be more beneficial if the device could detect the injured area and let patients know what is happening through the wearable. For example, P2 explained:

*If a device can detect which areas have been injured, it will be more beneficial because therapy depends on different sections of injury. And try to add options to let people know what to do. Because normal people are not educated enough to find the treatment.*

[P2, patient, man, Bangladesh]

However, despite the potential benefits, the cost of rehabilitation was an issue, and this applied to both countries. While wearables such as the SaeboGlove (Saebo, Inc) “are really good” (PT1, physiotherapist, woman, United Kingdom), they can be “prohibitively expensive” (PT3, physiotherapist, woman, United Kingdom) for patients who may want to use them at home. We also found that using technology to support rehabilitation caused discomfort and anxiety for some of the patients. For example, Bangladeshi physiotherapists mentioned that their patients thought that technology was too complicated or scary. This was echoed by the patients. For example, P3 said:

*When they diagnosed me, they applied many devices to me. I was so scared to see them. It’s like, why so much equipment? When they told me I must take the therapy, I remembered the diagnosis system. I again got scared. I prefer everything to be natural.*

[P3, patient, woman, Bangladesh]

## Discussion

### Principal Results

Our results highlight the impact of sociocultural factors on rehabilitation in Bangladesh. In particular, the family plays an important role in supporting patients, and through their involvement they may enable or hinder the treatment. Furthermore, people have personal preferences regarding physiotherapists’ gender, which can negatively impact the treatment if male patients do not want to engage with female physiotherapists. We also show differences in approaches to rehabilitation, with Bangladeshi physiotherapists focusing on individual movements that are necessary to build strength in the affected parts, and British physiotherapists favoring a more holistic approach that covers functional movements and considers patients’ mental well-being. Finally, our participants reported that COVID-19 exacerbated the challenges of home-based rehabilitation. During the height of the pandemic, physiotherapists were not able to access their patients’ homes, which resulted in limited access to rehabilitation, interrupted treatment, and increased costs.

Nevertheless, participants were optimistic about the potential of using wearable technologies at home, although they had concerns regarding the complexity and cost of such devices. Availability of affordable devices can be useful in low-resource regions like Bangladesh as well as in high-income regions such as the United Kingdom, considering the high cost of existing solutions. We have learned from our participants that any device intended to be used in the home would need to support and monitor hand and finger movements and provide feedback on their accuracy. More importantly, it would need to be affordable. Our results echo previous research that shows a simple, affordable wearable can be good enough to identify certain movements [18] and that such a device can be developed using cheap components [24-27]. However, technical requirements are only one aspect. The success of rehabilitation relies on consistent engagement [13], and that consistency means that the device should be suitable for home-based use to fit into patients’ lives.

### Sociotechnical Considerations: How to Fit ULR Technologies Into Everyday Life

#### Overview

While our results suggest that a wearable device could help with rehabilitation in home-based settings, they also highlight several sociotechnical challenges that need to be addressed first. Even the best technology can fail if the target users do not want or are unable to use it [30], and this is particularly important if it can (intentionally or not) challenge or affect cultural norms or religious customs [33,34]. Below we discuss the key trends identified in our data and conclude with a set of practical considerations for developing ULR technologies for low-resource settings.

#### Designing for Gendered Norms and Expectations

Our results showing that gendered expectations toward physiotherapists can limit patients’ access to treatment are in line with earlier work that shows differences in treatment based on patients’ gender [35]. Furthermore, Stenberg et al [36] consider gender to be a social construct that is shaped by norms and social context, which affects rehabilitation at every stage: from the experiences of physiotherapists and patients to how the care is accessed and provided. While a person’s religion in itself does not affect stroke rehabilitation [37], it does influence familial relationships and expectations, playing an important role in ULR. As such, any rehabilitation device or system – both its functionality and design – should consider the values and expectations of its target users and their families and needs to be acceptable to both patients and their caregivers. Finally, any new technologies introduced into the home, even with the best intentions, may encounter barriers related to the home environment (including issues with finding the right location) [38] and could potentially result in increased workload as they would need to be operated and maintained. Given that most informal caregivers in Bangladesh are women [11], these effects could also disproportionately affect them. Therefore, any home-based rehabilitation technologies need to take all the above factors into account.

#### Designing With Technological Literacy and Acceptance in Mind

We also identified some apprehension and discomfort related to technology use among patients and caregivers. At the same time, participants were open to try out new things, although they acknowledged their limited literacy. This echoes previous research on patients’ and physiotherapists’ experiences with technology [39-41]. For example, research on remote rehabilitation during the COVID-19 pandemic highlighted issues with technology literacy [40]. One way to address this issue could be through supplementary materials, such as videos [42]: when presented with a blended physiotherapy intervention that included home-based components, participants appreciated videos representing the exercise [43]. Another way could be through exposure to new digital technology. This could be done through exhibitions, online consumer rating websites, or user networks [39], or it could be done on an individual level. One of our participants mentioned being scared of various rehabilitation technologies (see P3’s quote in the Theme 4 section), but if the technology had been carefully introduced, the experience could have been less stressful. Research shows that human intermediaries (eg, health professionals and family members) can help people use novel technologies and make the experience of using them less intimidating [44].

In addition, to improve acceptance, the design needs to reflect target users’ values and culture [45-47]—an approach that has been taken when designing other types of rehabilitation technologies. For example, Villada Castillo et al [48] designed a virtual reality game for ULR among stroke survivors in Colombia that used cultural references and traditional Andean activities to make it more accessible to older participants. While it may be easier to design a game informed by cultural references than a wearable device, understanding users’ aesthetic preferences could help with adoption. For example, Wu and Munteanu [49] developed a wearable device for fall risk assessment in the form of a belt. Using a familiar object made participants more comfortable with technology and ensured regular engagement, although they did request different styles and designs. Similarly, in a study focused on designing wearables for Anishinaabe older adults with dementia from the Manitoulin region of Northern Ontario [50], participants did not like the “big and clumsy” prototype and suggested designing it so that it resembled familiar objects, such as bracelets. These examples suggest that making a simple ULR device that draws inspiration from contexts familiar to end users could make it more accessible and help to minimize literacy issues if it resembles familiar objects.

#### Designing for Different Approaches to Treatment

Third, we identified differences in physiotherapy practices and implications of different treatment approaches, which can be explained by limited resources and logistical issues related to delivering physiotherapy at home and accessing health care facilities [11,12]—all of which were exacerbated by the COVID-19 pandemic. However, the Bangladeshi physiotherapists’ focus on fundamental movements could make it easier to develop low-cost wearables that can recognize them [18,26]. It may also make integration of rehabilitation in everyday life easier, as the simple movements (and therefore any wearable device that supports them) do not require a lot of space or a complicated setup, although they may still require renegotiation of social relationships and additional care work [38]. This raises the question of who should be the target user for rehabilitation technologies: the patient who will use them or the informal caregivers who will help the patient put them on, use them, and maintain them? Ideally, the needs of both groups should be addressed.

#### Designing for Low-Resource Settings

Finally, while our focus was on low-cost wearables, “cost” in the context of rehabilitation technologies can be understood as “value for money” [39], especially when even the cheapest device may be too expensive for some Bangladeshi patients or not worth purchasing if the home environment or family situation do not afford regular use. As such, another point worth considering is device ownership—perhaps the device should be developed for physiotherapy settings with recommendations from both caregivers and physiotherapists, and physiotherapists could lend it to patients and provide at least minimal training to users and their families. Furthermore, having a rented device could work as an additional motivator and provide a sense of accountability, which may be necessary given low adherence to rehabilitation treatments [13,14].

#### Practical Considerations and Design Recommendations

Based on the above discussion, we highlight the following practical considerations and recommendations that will help designers and developers to create ULR devices for end users in the Global South and other low-resource settings: First, ensure the device is simple and easy to use so that patients and caregivers can operate it without a complex setup. Second, avoid procedures for use that require a significant effort and time investment on the side of the user. Third, identify the minimum required movements that would benefit the patient while still being relatively simple to execute. Fourth, in addition to functional requirements, do not overlook requirements such as maintenance, charging, and storage. All these steps add to the existing workload and could lead to nonuse and eventual abandonment if they do not align with target users’ daily routines. Fifth, use low-cost components that are good enough to recognize target movements (eg, flex sensors and an accelerometer can work well [18]); consider energy consumption and battery life. Sixth, when developing the device, engage users, especially women, in a co-design process to ensure the design and functionality of the device reflect their lived experiences and align with their sociocultural values. This will also help to come up with designs that are more contextually and culturally acceptable and less intimidating.

### Strengths and Limitations

The involvement of physiotherapists, patients, and caregivers was the strength of our study as it helped to identify the needs and opinions of a range of key stakeholder groups. The interviews with UK physiotherapists helped to compare physiotherapy practices and better understand the needs of delivering treatment in the home and what may and may not be possible in the Bangladeshi setting. Finally, our focus on Bangladesh and understanding the needs of our participants provide insights that could be beneficial when developing ULR technologies aimed at other Global South settings with limited resources and similar sociocultural considerations.

Due to COVID-19 mobility restrictions, we experienced difficulties with accessing participants and could not recruit as many stroke patients and informal caregivers as we initially aimed. To expand our participant pool, we decided to cover other types of conditions that also require ULR, which may have impacted our results. Furthermore, the experiences of the pandemic might have affected the way participants thought about home-based rehabilitation and their responses. However, given that we were interested in the general approach to ULR, patient experiences with home-based rehabilitation, and the role and concerns of caregivers, the results still provide relevant insights as participants were asked to describe their real experiences. As discussed in the Results (in the Theme 3 section), participants openly shared their COVID-19 experiences and how their rehabilitation was affected by the pandemic, which we took into account when forming the practical considerations.

We interviewed 14 participants in total. We acknowledge that the data cannot be generalized, but the sample size is typical for an in-depth formative study (see, for example, Stawarz et al [51,52]) and is sufficient to identify key design considerations [53] and provide a further understanding of the complexities and social and economic context of home-based ULR. Following the person-centered approach [29], the next step in our research program is to organize in-depth design workshops with a larger number of poststroke patients and their formal and informal caregivers and to develop demonstrator prototypes that can be tested in their homes to gather further insights.

### Conclusions

A qualitative study with physiotherapists, patients, and caregivers focused on their experiences helped us to identify several sociocultural challenges and considerations that should be taken into account when developing ULR technologies for the home in low-income countries. While it is possible to build a low-cost wearable device for ULR, these sociotechnical challenges need to be considered together with functional requirements, as interpersonal relationships involving patients, physiotherapists, and caregivers (and other family members) can affect access to and quality of care.

## Abbreviations

ULR: upper-limb rehabilitation
